# Evaluating Retrieval Augmented Generation-enhanced Large Language Models for Question Answering On German Neurovascular Guidelines

**DOI:** 10.1007/s00062-025-01562-z

**Published:** 2025-09-02

**Authors:** Marius Vach, Michael Gliem, Daniel Weiss, Vivien Lorena Ivan, Frederik Hauke, Christian Boschenriedter, Christian Rubbert, Julian Caspers

**Affiliations:** 1 Department of Diagnostic and Interventional Radiology, Medical Faculty and University Hospital Düsseldorf, Heinrich-Heine-University Düsseldorf, Moorenstraße 5, 40225 Düsseldorf, Germany; 2 Department of Neurology, Medical Faculty and University Hospital Düsseldorf, Heinrich-Heine-University Düsseldorf, Düsseldorf, Germany

**Keywords:** Artificial intelligence, Large language model, Neurovascular disease, Ischemic stroke, Carotid stenosis, Medical guidelines, Evidence-based medicine

## Abstract

**Purpose:**

To investigate the feasibility of Retrieval-augmented Generation (RAG)-enhanced Large Language Models (LLMs) in answering questions about two German neurovascular guidelines.

**Methods:**

Four LLMs (GPT-4o-mini, Llama 3.1 405B Instruct Turbo, Mixtral 8 × 22B Instruct, and Claude 3.5 Sonnet) with RAG as well as GPT-4o-mini without RAG were evaluated for generating answers about two German neurovascular guidelines (“S3 Guideline for Diagnosis, Treatment, and Follow-up of Extracranial Carotid Stenosis” and “S2e Guideline for Acute Therapy of Ischemic Stroke”). The answers were classified as “correct”, “inaccurate”, or “incorrect” by two neurovascular experts in consensus. Additionally, retrieval performance of five retrieval strategies was analyzed on a synthetic dataset of 384 questions.

**Results:**

Claude Sonnet 3.5 achieved the highest answer correctness (70.6% correct, 10.6% wrong), followed by Llama 3.1 (64.7%, 15.3% wrong), GPT-4o-mini with RAG (57.6%, 15.3% wrong), and Mixtral (56.6%, 17.6% wrong). GPT-4o-mini without RAG performed significantly worse (20.0%, 32.9% wrong). Retrieval errors were the primary cause of incorrect answers (80%). For retrieval, BM25 achieved the highest accuracy (82.0%), outperforming vector-based methods like “BAAI/bge-m3” (78.4%).

**Conclusion:**

RAG significantly improves LLM accuracy for medical guideline question answering compared to the inherent knowledge of pretrained LLMs alone while still showing significant error rates. Improved accuracy and confidence metrics are needed for safer implementation in clinical routine. Additionally, our results demonstrate the strong performance of general LLMs in medical question answering for non-English languages, such as German, even without specific training.

**Supplementary Information:**

The online version of this article (10.1007/s00062-025-01562-z) contains supplementary material, which is available to authorized users.

## Introduction

Medical guidelines are a relevant cornerstone for health care and play a crucial role in standardizing patient care and improving medical decision making. These guidelines, developed through extensive expert consensus, provide evidence-based recommendations for diagnosis, treatment, and management of medical conditions. However, as medical knowledge expands and guidelines become increasingly comprehensive, physicians face challenges in quickly accessing and interpreting specific information relevant to their immediate clinical decisions, which presents a major barrier to guideline adherence [1, 2].

Recent advances in artificial intelligence, specifically Large Language Models (LLMs), have shown promise in processing and interpreting complex medical text [3–5]. However, a critical challenge with LLMs is their tendency to fabricate facts—a phenomenon called “hallucination”—which is particularly concerning in medical contexts [6]. Retrieval-augmented Generation (RAG) offers a solution by enabling these models to ground their responses in specific documents rather than relying solely on their pre-trained knowledge [5]. This approach first identifies relevant sections of documents using retrieval algorithms, then uses these sections as context to generate detailed and specific answers to queries, making it particularly valuable for medical applications where accuracy and reliable sourcing of information are paramount [5].

This study evaluates the feasibility of RAG for question answering on German medical guidelines. In particular, the performance of different LLMs in answering clinically demanding questions about guidelines is evaluated using the example of two relevant German neurovascular guidelines, i.e. the German “S3 Guideline for Diagnosis, Treatment, and Follow-up of Extracranial Carotid Stenosis” and the German “S2e Guideline for Acute Therapy of Ischemic Stroke”. We first established baseline performance using a high-quality embedding model to assess LLM generation capabilities, then investigated alternative retrieval approaches after identifying retrieval errors as the primary source of incorrect answers.

Our findings could help inform the development of artificial intelligence (AI) assisted clinical decision support tools for medical guideline interpretation, potentially improving the accessibility and practical application of medical knowledge in clinical settings and, thus, standardized patient care.

## Methods

The local ethics committee waived the need for approval for this study.

### Knowledge Base

The German “S3 Guideline for Diagnosis, Treatment, and Follow-up of Extracranial Carotid Stenosis” (version 2.1, accessed through awmf.org) and the German “S2e Guideline for Acute Therapy of Ischemic Stroke” (version 5.1, accessed through awmf.org) were evaluated. For the analyses, the content of the guidelines was split into chunks with defined sizes. Using the ‘SentenceSplitter’ algorithm from the python package ‘llama-index’ (version 0.11.1), the PDF documents were processed into a combined total of 770 chunks (490 chunks for the S3 Carotid Stenosis guideline and 280 chunks for the S2e Ischemic Stroke guideline), with a maximum size of 1024 characters per chunk. This algorithm creates chunks that respect sentence boundaries while approaching the character limit, resulting in variable-sized chunks that optimize semantic coherence.

### Retrieval-augmented Generation

RAG was applied to enable question answering of LLMs informed by retrieved document content of medical guidelines. For this, relevant text chunks for a certain query were retrieved from the 770 extracted guideline chunks using text-embedding based retrieval.

Generally, text embedding-based retrieval operates in three steps: First, a large language model converts text chunks into vector representations that capture their semantic meaning. Secondly, the same model converts the search query or question into a vector representation with the idea that questions and relevant text chunks are similar to each other in vector space. To retrieve the most similar text chunks, the system calculates the cosine similarity between the query vector and each text chunk vector, returning the top‑n most similar chunks. For the main analysis, OpenAI’s “text-embedding-3-large” was used as embedding model.

Then the relevant text chunks were provided to the LLM as context and the LLM was prompted to answer the query. The concrete prompt can be found in the supplemental material.

The RAG pipeline was implemented in Python using the “llama-index” library (version 0.11.1). We selected the top‑5 text chunks as context based on both standard RAG implementation practices and practical considerations. This configuration provides approximately 4000 tokens of context, which is sufficient to capture relevant medical information while minimizing the risk of including irrelevant or contradictory information that could confuse the model. While increasing the number of retrieved chunks might improve information recall, it would also increase inference time, computational costs, and potentially introduce noise that could degrade answer quality.

### Large Language Models Evaluated

The retrieved text chunks were provided to a language model, which answered the questions based on this contextual information.

Four different large language models were evaluated in their ability to answer questions on the two medical guidelines: GPT-4o-mini (OpenAI, San Francisco, California, USA), Llama 3.1 405B Instruct Turbo (Meta, Menlo Park, California, USA), Mixtral 8 × 22B Instruct (Mistral AI, Paris, France) as well as Claude 3.5 Sonnet (Anthropic, San Francisco, California, USA). To minimize output variability in model responses, the temperature parameter was set to 0 for all models.

### Question-answering Evaluation

To evaluate the correctness of the LLM-provided answers a German benchmark dataset of 85 questions concerning medical care in the context of the two neurovascular guidelines were created by an expert neuroradiologist (JC, 12 years of experience) and a radiologist (MV, 6 years of experience). These questions were then answered by the different LLMs using the previously described RAG process. We used “text-embedding-3-large” by OpenAI as the embedding model. Additionally, we used GPT-4o-mini without a retrieval step to answer the questions as a non-RAG control. The question set has been released on HuggingFace (https://huggingface.co/datasets/rasmus1610/neurovascular-qa).

The resulting answers of each RAG pipeline as well as GPT-4o-mini without retrieval were then independently rated by one expert neuroradiologist (JC, board-certified, 12 years of experience, native German speaker) and one expert neurologist specializing in neurovascular care (MG, board-certified, 18 years of experience, native German speaker) using a standardized clinical accuracy framework. Evaluators assessed answers based solely on clinical validity and safety implications, not writing quality or style. The three-point rating scale was defined by specific clinical criteria:

- Correct: The medical recommendation was clinically sound and accurate, with no meaningful errors that would affect patient care decisions. Minor inconsequential reasoning errors were permitted if they didn’t impact clinical interpretation.
- Inaccurate: The answer contained incomplete information, minor errors, or logical inconsistencies that might cause confusion but wouldn’t lead to harmful clinical decisions.
- Wrong: The response failed to address the question, contained factually incorrect information, was critically incomplete, or included potentially dangerous clinical recommendations.

**Fig. 1:**
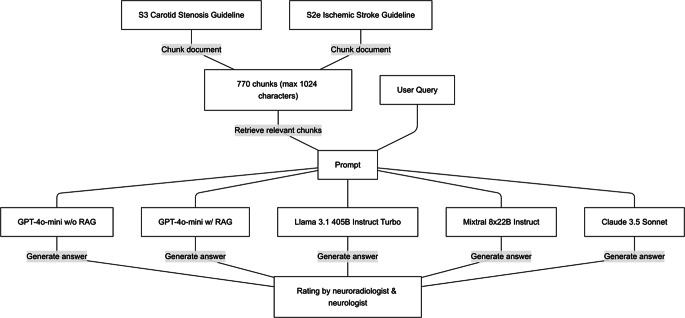
This diagram illustrates the process of answering clinical questions using RAG-enhanced LLMs. German neurovascular guidelines were split into 770 text chunks. For each query, top‑5 relevant chunks were retrieved using OpenAI’s text-embedding-3-large, then provided to four LLMs (GPT-4o-mini, Llama 3.1, Mixtral, Claude Sonnet 3.5) to generate answers. Responses were evaluated by two experts (neuroradiologist and neurologist) on correctness, accuracy, and safety

Evaluators were blinded to model identity throughout the assessment process. After independent rating of all 85 questions, any disagreements were resolved through discussion to reach clinical consensus. The evaluation focused on whether a clinician following the recommendation would make appropriate clinical decisions based on current guideline standards. A flow chart of the question answering process can be found in Fig. 1.

All incorrect answers from each model were re-evaluated by one author (<anonymized>) to identify sources of incorrect answering. The reasons for incorrect answers were categorized into three classes: “retrieval”, “reasoning” and “question phrasing.” Retrieval problems occurred when the RAG pipeline failed to retrieve contextually relevant information that directly addressed the query. Reasoning problems resulted from the model’s failure to interpret, synthesize, or logically infer answers from adequate context. Question phrasing problems stemmed from ambiguous, overly broad, or poorly structured questions.

### Retrieval Evaluation

To evaluate the retrieval step further, four text embedding models (OpenAI “text-embedding-3-small” (OpenAI Small), OpenAI “text-embedding-3-large” (OpenAI Large), “sentence-transformers/distiluse-base-multilingual-cased-v1” (HF1), and “BAAI/bge-m3” (HF2)) were evaluated alongside the keyword-based retrieval strategy BM25 [7]. BM25 is a keyword-based text search algorithm based on the well-known TF-IDF (“term frequency/inverse document frequency”) algorithm [8].

A synthetic dataset was created to evaluate the information retrieval methods: 384 text chunks were randomly sampled from the two guidelines, and a LLM (Meta Llama 3.1 405B Instruct Turbo, (Meta, Menlo Park, California, USA)) was prompted to generate a question answerable with each specific chunk. The concrete prompt can be found in the supplemental material. The dataset has been released on HuggingFace (https://huggingface.co/datasets/rasmus1610/neurovascular-qa).

**Fig. 2:**
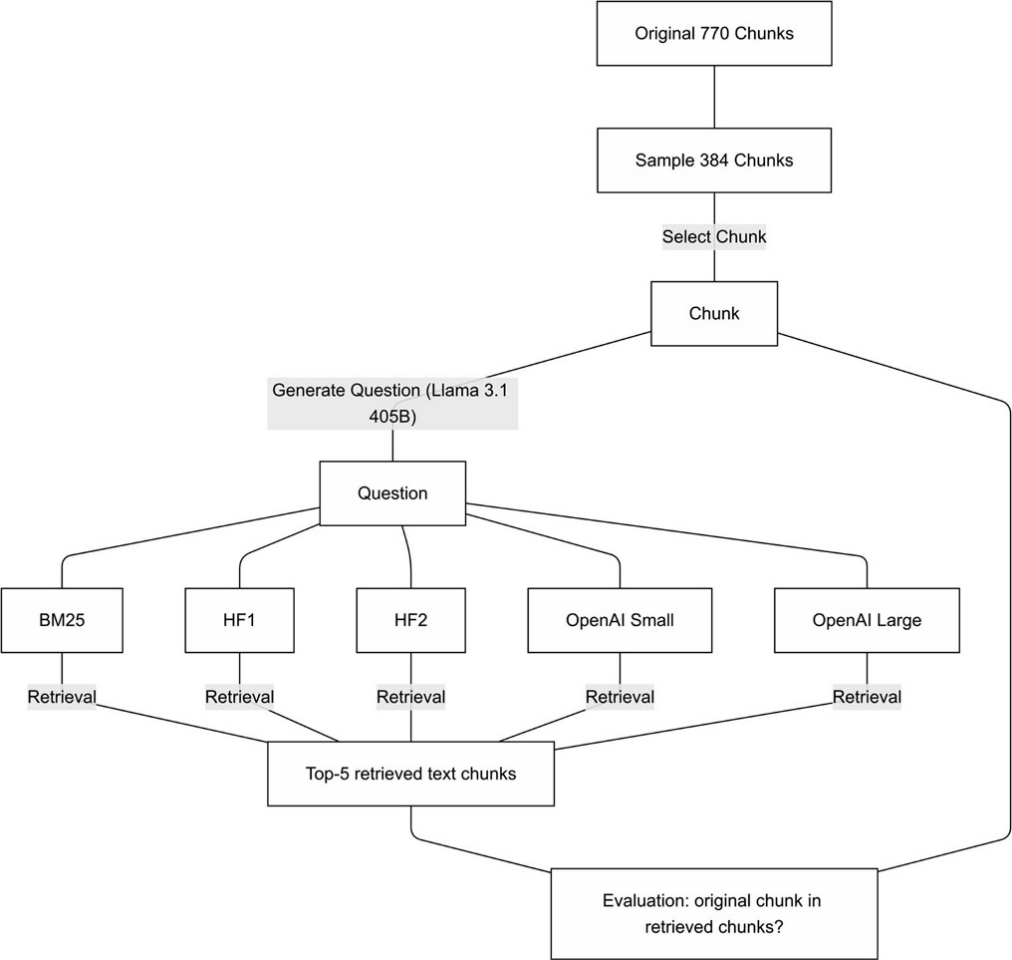
To assess retrieval performance, a synthetic dataset of 384 questions was created by sampling guideline chunks and generating queries with Llama 3.1 405B. Five retrieval methods (BM25, HF1, HF2, OpenAI Small/Large) were compared based on their ability to retrieve the original chunk in the top‑5 results. This analysis identified BM25 as the most effective strategy for this task (*HF1* sentence-transformers/distiluse-basemultilingual-cased-v1; *HF2* BAAI/bge-m3; *OpenAI Small* text-embedding-3-small; *OpenAI Large* text-embedding-3-large)

The retrieval strategies were then tested by measuring how frequently the original text chunk appeared among the top‑5 results retrieved for its corresponding question using the technique described above. A flow chart of the retrieval evaluation process can be found in Fig. 2.

### Code Implementation

The retrieval-augmented generation pipeline was implemented using the Python programming language (version 3.12.7) as well as the “llama-index” library (version 0.1.1). The proprietary LLMs were used through their respective proprietary application programming interfaces (APIs). The two open-weights LLMs (Llama 3.1 405B and Mixtral 8 × 22B) were used through an application programming interface (API) provided by Together.ai (San Francisco, California, USA). The code can be found in the following GitHub repository: https://github.com/AInII-Lab/nv-rag.

### Statistical Analysis

Proportions of correct, inaccurate and incorrect answers are given as percentages with the 95% confidence interval. Correctness proportions were compared across models using the Kruskal-Wallis-Test and post-hoc pairwise comparisons using Chi-Square-Tests. Resulting *p*-values were corrected for multiple comparisons using the Bonferroni method. Confidence intervals were calculated using the Wilson method. All statistical analyses were performed using Python (version 3.12.7) and the “scipy” (version 1.14.0), “pandas” (2.2.2) and “numpy” (1.26.4) packages.

## Results

### Question Answering Results

The performance in RAG-based question answering of four large language models was evaluated on a test set of 85 questions by a neuroradiologist and neurologist, with responses categorized as “correct,” “inaccurate,” or “wrong” in consensus. Claude Sonnet 3.5 achieved the highest accuracy with 70.6% correct responses (95% CI: [60.9%, 80.3%]), followed by Llama 3.1 405B at 64.7% (95% CI: [54.5%, 74.9%]), GPT-4o-mini with RAG at 57.6% (95% CI: [47.0%, 68.2%]), and Mixtral 8 × 22B at 56.6% (95% CI: [46.0%, 67.2%]). GPT-4o-mini without RAG performed significantly worse, with only 20.0% correct responses (95% CI: [11.4%, 28.6%]), 32.9% wrong responses (95% CI: [23.9%, 43.5%]), and 47.1% inaccurate responses (95% CI: [36.8%, 57.6%]). The results can be found in Table 1. The Kruskal-Wallis-Test showed a statistically significant difference in the performance among the different LLMs (*p* < 0.001). Pairwise Chi-square tests revealed no statistically significant differences between any pair of the four top-performing models (all *p* > 0.05). However, GPT-4o-mini without RAG showed statistically significant differences when compared to all other models (each *p* < 0.001 after Bonferroni correction). Notably, the addition of RAG to GPT-4o-mini substantially improved its performance, nearly tripling the percentage of correct responses while reducing both inaccurate (47.1 to 27.1%) and wrong (32.9 to 15.3%) responses.

**Table 1:** Performance of different Large Language Models (LLMs) on medical question answering as evaluated by two expert physicians.

Model	Wrong	Inaccurate	Correct
GPT-4o-mini w/o RAG	28 (32.9%)	40 (47.1%)	17 (20.0%)
GPT-4o-mini w/RAG	13 (15.3%)	23 (27.1%)	49 (57.6%)
Llama 3.1	13 (15.3%)	17 (20.0%)	55 (64.7%)
Mixtral	15 (17.6%)	22 (25.9%)	48 (56.6%)
Claude Sonnet 3.5	9 (10.6%)	16 (18.8%)	60 (70.6%)

**Fig. 3:**
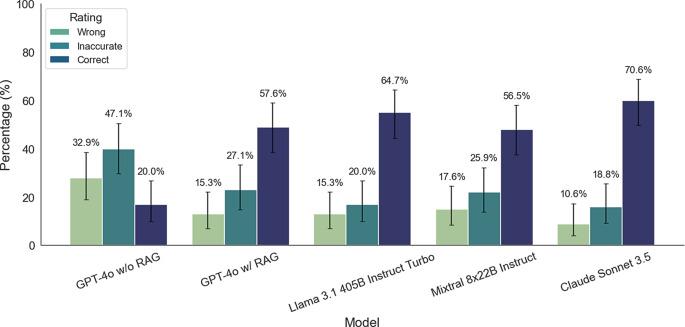
Question answering performance of the different large language models on 85 test questions, evaluated by a neuroradiologist and neurologist in consensus

Additionally, we performed two separate binary comparisons using Kruskal-Wallis tests: (1) correct vs. combined inaccurate/wrong responses showed no significant difference (*p* = 0.46), while (2) wrong vs. combined correct/inaccurate responses revealed a significant difference (*p* = 0.01). A pairwise comparison afterwards identified a statistical difference in the proportion of wrong answers between GPT-4o-mini w/o RAG and Claude Sonnet 3.5 after Bonferroni correction (*p* = 0.003). A bar chart with the question answering performance can be found in Fig. 3.

### Analysis of Wrong Answers

Incorrect answers were categorized into three types based on the answer text and retrieved context: Retrieval errors (80%) were the dominating source of error, primarily due to insufficient specificity in retrieved contexts (e.g., by confusing carotid endarterectomy and stenting procedures), structural data loss (e.g., unreadable tables), and fragmented information sources. Reasoning failures (16%) derived from model misinterpretation of valid context, such as misreading age thresholds for hemicraniectomy, or hallucinations when extrapolating beyond retrieved data. Among all models tested, only Claude 3.5 Sonnet achieved zero reasoning failures. Question phrasing (4%) contributed minimally but highlighted vulnerabilities to ambiguous phrasing (e.g., “In which cases is dual platelet inhibition advisable?”, lacking clinical specificity). These results identify retrieval as the primary bottleneck, with semantic mismatches critically impairing answer quality. Table 2 shows the different problem categories per model.

**Table 2:** Problems leading to wrong answers categorized per model.

Model	Problem Category	Count
GPT-4o-mini	Retrieval	10 (77%)
	Reasoning	3 (23%)
	Question phrasing	0 (0%)
Llama 3.1 405B Instruct Turbo	Retrieval	11 (85%)
	Reasoning	2 (15%)
	Question phrasing	0 (0%)
Mixtral 8 × 22B Instruct	Retrieval	11 (73%)
	Reasoning	3 (20%)
	Question phrasing	1 (7%)
Claude Sonnet 3.5	Retrieval	8 (89%)
	Reasoning	0 (0%)
	Question phrasing	1 (11%)

### Retrieval Results

**Fig. 4:**
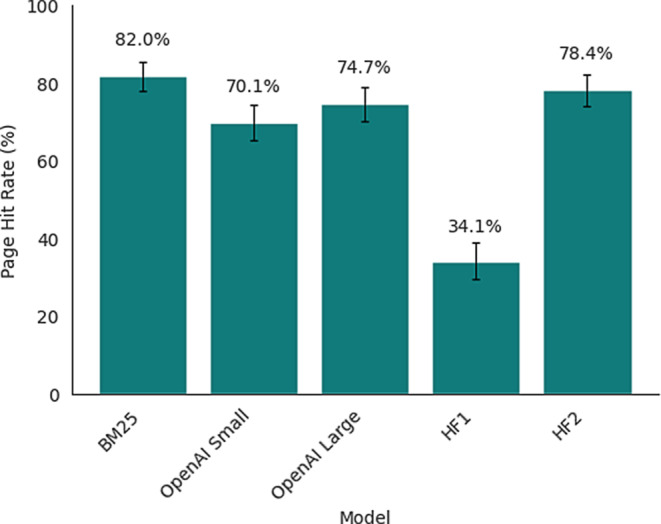
Performance of the different retrieval methods measured as the proportion the correct page from the guidelines was retrieved (*HF1* sentence-transformers/distiluse-base-multilingual-cased-v1; *HF2* BAAI/bge-m3; *OpenAI Small* text-embedding-3-small; *OpenAI Large* text-embedding-3-large)

To further analyze retrieval errors as the primary cause of incorrect answers, we evaluated five different retrieval strategies on a synthetic test set of 384 questions. BM25 achieved the highest retrieval accuracy (82.0%, 95% CI [77.9%, 85.5%]), followed by HF2 (78.4%, [74.0%, 82.2%]) and OpenAI Large (74.7%, [70.2%, 78.8%]). OpenAI Small performed moderately (70.1%, [65.3%, 74.4%]), while HF1 showed significantly lower performance (34.1%, [29.6%, 39.0%]). Pairwise comparisons with Bonferroni correction revealed that BM25 significantly outperformed OpenAI Small (*p* < 0.001) and HF1 (*p* < 0.001) but showed no significant difference to OpenAI Large or HF2. HF1’s performance was significantly lower than all other models (all *p* < 0.001). A bar chart of the retrieval performance can be found in Fig. 4.

## Discussion

The aim of this work was to evaluate different approaches for question answering on medical guidelines using RAG. Our analysis revealed comparable performances of RAG-based approaches using different LLMs, but a striking performance gain of RAG compared to non-RAG-based GPT-4o-mini usage. Additionally, we identified failure to retrieve relevant context as the main reason for wrong answers and analyzed different retrieval strategies on a synthetic dataset of 384 questions.

On the question answering task, the performance gap between GPT-4o-mini without RAG-based guideline access (20.0% correct) and with guideline access using RAG (57.6% correct) demonstrates the crucial importance of grounding model responses in the actual guideline text rather than relying on the model’s inherent pre-trained knowledge to avoid hallucinations and provide factually correct answers, especially in critical contexts like healthcare. This finding is in line with previous publications, which also identified a substantial improvement in accuracy for medical question answering when grounding the LLM on medical literature using RAG [5, 9, 10], but only investigated English questions and sources. This technique also ensures that recommendations are based on up-to-date guidelines rather than potentially outdated AI knowledge, and allows the system to adapt as medical guidelines are updated.

Among all tested models, Claude Sonnet 3.5 performed best with 70.6% correct answers and only 10.6% wrong answers. Additionally, Claude Sonnet was the only model that did not commit reasoning errors, i.e. hallucinations, on retrieved content. However, rates of incorrect or inaccurate responses of this best-performing model were still 29.4% in total, which highlights that current AI systems, while promising, are not yet reliable enough for unsupervised clinical use. The significant error rates indicate that these systems could only assist, not replace, clinical decision-making guided by expert knowledge and it requires users to have sufficient expertise to identify system errors. Furthermore, more sophisticated techniques for developing specialized models, e.g., using additional fine-tuning, might be needed rather than using RAG-enhanced LLMs out of the box to enable suitability for clinical applications. Nonetheless, the current results clearly highlight the potential of such RAG systems even with their current performance, e.g., for assisting lookup of references or for educational purposes.

Our analysis revealed that retrieval problems were the primary cause of incorrect answers (80% of cases). In contrast, reasoning problems, where the large language model (LLM) misinterpreted the correctly retrieved context, were comparatively rare (16%). This highlights the strong performance of modern LLMs and indicates a clear path forward for improving these types of systems. Our results indicate that when developing reliable clinical decision support systems, focus should be placed on improving retrieval strategies, since most large language models perform similarly in generating answers from relevant context.

Our evaluation of different retrieval approaches showed consistently high performance, with the top four systems achieving accuracies between 82.0 and 70.1%. Interestingly, the traditional keyword-based BM25 retrieval system outperformed more complex vector-based approaches (82.0% vs 78.4% for HF 2). Since many medicine-specific words are not in the vocabulary of the LLM, there are probable difficulties for the model in understanding their semantic meaning and creating fitting vector embeddings. BM25 searches for the keywords directly and is not dependent on a specific vocabulary. But it also suggests that for specialized medical guidelines with clear terminology, simple and computationally efficient retrieval methods may be sufficient. This is particularly relevant for potential clinical implementations, as BM25 offers a reliable, transparent, and resource-efficient retrieval solution that could easily be run on-premise without the need to send sensitive clinical data to external services.

A particularly interesting finding in this regard is the strong performance of Llama 3.1 405B (64.7% correct responses), showing no statistical difference from the best-performing model, Claude Sonnet 3.5 (70.6%). This result has important implications for healthcare data privacy and practical clinical implementation when developing comparable clinical decision support systems using sensitive patient data. Unlike commercial AI services, Llama 3.1 is an open-weights LLM and can be deployed locally within hospital networks. This addresses a major barrier to AI adoption in healthcare: the need to share potentially sensitive data with external commercial API services. The fact that Llama 3.1 achieves comparable performance to cloud-based models while maintaining data sovereignty makes it particularly attractive for healthcare institutions bound by “General Data Protection Regulation (GDPR)” and other privacy regulations. While setting up local AI systems requires technical resources, the combination of strong performance and data privacy makes this a potentially worthwhile endeavor.

For the current study, we used guidelines and questions in German language. However, the language models used in the current analysis were primarily trained on English data and no models specifically fine-tuned to the German language were used. While modern LLMs typically support multiple languages, their training data and medical content evaluations are predominantly in English. Additionally, most of the studies examining RAG on medical content are conducted with English text [4, 5]. Nevertheless, our results demonstrate these models’ strong comprehension of German medical texts and provides solid results for question answering even in a non-preferred language of current LLMs.

Our work comes with some limitations. We only evaluated the performance on two neurovascular guidelines, which hinders the generalizability of the results to other areas of medicine. Nonetheless the results provide interesting information on the ability of neural networks and retrieval mechanisms to answer specific medical questions in other languages than English, e.g., German in this case.

Additionally, this was no exhaustive evaluation of retrieval methods and LLMs for medical question answering, as there are many more retrieval methods and LLMs out there. This work concentrated on the most popular LLMs and classic retrieval mechanisms. The quite comparable results between the tested models indicate that the concrete choice of the model might not be that important, but that RAG technology is a relevant factor to provide a fundamental knowledge basis for question answering. However, modern techniques like late interaction retrieval or hybrid search may be addressed in future research.

Furthermore, innovations like “prompt caching”, available through Anthropic’s API, combined with longer context windows of LLMs, may make retrieval-augmented generation obsolete for question answering over a limited number of documents in the future. Additionally, new retrieval mechanisms like “Hybrid search” or “Late interaction retrieval” may lead to better retrieval performance [11]. Evaluating these newer approaches will be a topic for future research.

Additionally, our study used a fixed context window of 5 text chunks for the RAG pipeline, which represents a practical balance between information completeness and computational efficiency. While this approach follows common RAG implementation practices, the optimal number of retrieved chunks may vary depending on the complexity of medical questions and the structure of the guidelines. Future research should investigate how different context window sizes affect the performance of medical RAG systems, particularly for highly specialized clinical questions requiring synthesis of information from multiple guideline sections.

In conclusion, our study demonstrates both the potential and current limitations of AI systems in medical guideline interpretation. While the best-performing models achieved 70.6% accuracy in answering guideline-specific questions correctly, the significant error rate indicates that using these systems out-of-the-box is not yet suitable for unsupervised clinical use. However, the strong performance of open-weights models like Llama 3.1 405B, combined with efficient retrieval methods like BM25, suggests a practical path toward privacy-compliant AI assistance in clinical settings using RAG approaches. These systems could serve as valuable knowledge bases for rapid guideline information access, particularly beneficial for emergency settings or less experienced clinicians, while always maintaining human oversight. Future research should focus on improving response accuracy and developing reliable confidence metrics to support safer clinical implementation.

## Supplementary Information

Concrete prompts used for question answering and synthetic question generation

## Abbreviations

AI: Artificial Intelligence
API: Application Programming Interface
GDPR: General Data Protection Regulation;
GPT: Generative Pre-trained Transformer
LLM: Large Language Model
RAG: Retrieval-augmented Generation
TF-IDF: Term frequency-inverse document frequency
