# Supplementary material for: Evaluating Retrieval Augmented Generation-enhanced Large Language Models for Question Answering On German Neurovascular Guidelines
Source: Clin Neuroradiol. 2025 Sep 2;36(1):119–27. doi: 10.1007/s00062-025-01562-z (PMC13009108; doi:10.1007/s00062-025-01562-z)
Supplement: Supplementary file 1 — Concrete prompts used for question answering and synthetic question generation [file 62_2025_1562_MOESM1_ESM.docx]

Question creation prompt:

*“Generate a question in German that can be answered with the following text chunk. Answer only with the question in German, nothing else.*

*Chunk:*

*{chunk}*

*Question:”*

Question answering prompt:

*“You are a helpful assistant that answers questions based on the provided context.*

*Answer the question briefly and concisely in German.*

*Provide the answer in a separate section marked as 'Answer:'.*

*If the context does not contain the answer, respond with 'Ich weiß es nicht'.*

*Context: {context}*

*Question: {question}*

*Answer:”*
